# Distribution of Viral Respiratory Infections during the COVID-19 Pandemic Using the FilmArray Respiratory Panel

**DOI:** 10.3390/biomedicines10112734

**Published:** 2022-10-28

**Authors:** Ying-Ju Chen, Tze-Kiong Er

**Affiliations:** 1 Division of Laboratory Medicine, Asia University Hospital, Asia University, Taichung 413, Taiwan; 2 Department of Medical Laboratory Science and Biotechnology, Asia University, Taichung 413, Taiwan; 3 Department of Nursing, Asia University, Taichung 413, Taiwan

**Keywords:** FilmArray respiratory panel, respiratory viral pathogens, COVID-19, coinfection

## Abstract

This study was conducted to evaluate the distribution of respiratory viral pathogens in the emergency department during the coronavirus disease 2019 (COVID-19) pandemic. Between May 2020 and September 2022, patients aged between 0.1 and 98 years arrived at the emergency department of Asia University Hospital, and samples from nasopharyngeal swabs were tested by the FilmArray^TM^ Respiratory Panel (RP). SARS-CoV-2 positivity was subsequently retested by the cobas Liat system. There were 804 patients for whom the FilmArray^TM^ RP was tested, and 225 (27.9%) of them had positive results for respiratory viruses. Rhinovirus/enterovirus was the most commonly detected pathogen, with 170 (61.8%) cases, followed by adenovirus with 38 (13.8%), SARS-CoV-2 with 16 (5.8%) cases, and coronavirus 229E, with 16 (5.8%) cases. SARS-CoV-2 PCR results were positive in 16 (5.8%) cases, and there were two coinfections of SARS-CoV-2 with adenovirus and rhinovirus/enterovirus. A total of 43 (5.3%) patients were coinfected; the most coinfection was adenovirus plus rhinovirus/enterovirus, which was detectable in 18 (41.9%) cases. No atypical pathogens were found in this study. Intriguingly, our results showed that there was prefect agreement between the detection of SARS-CoV-2 conducted with the cobas Liat SARS-CoV-2 and influenza A/B nucleic acid test and the FilmArray^TM^ RP. Therefore, the FilmArray^TM^ RP assay is a reliable and feasible method for the detection of SARS-CoV-2. In summary, FilmArray^TM^ RP significantly broadens our capability to detect multiple respiratory infections due to viruses and atypical bacteria. It provides a prompt evaluation of pathogens to enhance patient care and clinical selection strategies in emergency departments during the COVID-19 pandemic.

## 1. Introduction

Severe acute respiratory syndrome coronavirus 2 (SARS-CoV-2) spread rapidly worldwide by the end of 2019. SARS-CoV-2, first detected in Wuhan city, Hubei province, has now infected over one billion people, causing over two million deaths globally [1]. Multiple studies have shown the epidemiology of viral infection during the COVID-19 pandemic [2,3,4,5], and coexisting respiratory pathogens among COVID-19 patients [6,7,8,9,10]. The epidemiology of respiratory pathogens in regions with high prevalence may be different from that in regions with low prevalence of COVID-19. Recently, Huang CP et al. conducted a comprehensive study of respiratory pathogens in a Taiwanese population using multiplex PCR [3]. They showed that 59.7% of identified organisms were bacteria, and viruses accounted for 23.9%. Notably, several case studies showed that a few patients were coinfected with SARS-CoV-2 and influenza virus [11,12], and with common bacterial pathogens [13,14]. Feldman C et al. indicated that coinfections and/or superinfections in patients with COVID-19 infections seemed to be related to the severity of COVID-19 infection and prognosis [15].

Acute respiratory infection (ARI) is a public health concern and is also the major cause of outpatient visits and hospitalizations in all age categories [16,17]. It is caused by a different group of pathogens that affect the human airways. Additionally, it is a life-threatening and the second leading cause of death for children under 5 years of age [18,19]. Approximately 80% of the total worldwide cases of ARI occur in Southeast Asia, followed by sub-Saharan African countries [20]. Respiratory viruses, including adenovirus (ADV), rhinovirus (RV), respiratory syncytial virus (RSV), and influenza viruses, are the major causes of ARIs in children. Additionally, atypical pathogens are major causes of pediatric respiratory infections. For example, *Mycoplasma pneumoniae* (*M. pneumoniae*) is one of the most frequent atypical pathogens, and approximately 10–40% of hospitalized children have community-acquired pneumonia [21]. Additionally, *M. pneumoniae* is a pivotal pneumonia-causing pathogen accounting for 10–30% of community-acquired pneumonia cases in the pediatric population in Taiwan [22]. The early diagnosis of the organism is helpful for the accurate selection of suitable medication, which can improve the clinical care of patients and prevent the overuse or even abuse of antibiotics [17]. In addition, the early detection of contagious pathogens can allow for the early isolation of specific individuals, thus mitigating the outbreak of pathogens. 

Traditional PCR has been applied to identify pathogens for many years. However, the limitations of traditional PCR are that it can only target a single pathogen, and it also has a high risk of contamination due to the handling process. Therefore, it is not considered completely satisfactory for clinical use, especially for emergency departments, because the turnaround time is critical. In recent years, these problems have been overcome due to several multiplex platforms using PCR, and assays for nucleic acid amplification for the simultaneous identification of two or more viruses have been established. PCR methods, especially multiplex real-time polymerase chain reaction (RT-PCR) techniques, have been applied in the laboratory to provide the high-speed detection of multiple respiratory organisms from patient specimens in an easy workflow [23]. In addition, an expanded range of viral and bacterial targets can be differentiated and identified by PCR assay. There are also several multiplex PCR platforms available with the potential to identify multiple pathogens in a single reaction [24,25,26,27]. Nucleic acids of viral or bacterial pathogens in a single reaction detected by multiplex panels are being increasingly applied for the detection of multiple respiratory infections. In fact, emergency physicians encountered long turnaround times and delayed therapy when treating possible viral respiratory diseases before the widespread usage of molecular testing. The application of high-speed molecular testing has markedly enhanced identification by detecting multiple pathogens simultaneously, increasing sensitivity and specificity, decreasing time to positivity, and eventually reducing time to clinical treatment [28]. 

As previously mentioned, coinfections with one or more additional pathogens in COVID-19 patients may have a poor disease outcomes. Therefore, it is important to understand the frequency of coinfection with other respiratory organisms, and the profile of organisms can contribute to select appropriate treatment. The aim of this study was to evaluate the distribution of viral respiratory pathogens in central Taiwan during the COVID-19 pandemic.

## 2. Materials and Methods

### 2.1. Study Design and Data Collection

In the present study, we retrospectively analyzed the results of all the samples and aimed to understand the prevalence of each respiratory organism, which was analyzed during a 1.5-years period (May 2021 to September 2022) using the FilmArray^TM^ RP (BioFire® Diagnostics, Salt Lake City, UT, USA). A total of 804 nasopharyngeal swab (NPS) specimens were obtained from patients suspected of suffering from respiratory infection. Among 804 respiratory samples tested, 365 (45.4%) were from male patients and 439 (54.6%) were from female patients. The median age of these 804 patients was 6 years (0.1–98).

### 2.2. FilmArray^TM^ Respiratory Panel v2.1 Testing

The FilmArray^TM^ RP (BioFire® Diagnostics, Salt Lake City, UT, USA) is a multiplex PCR assay and that is also fully automated. It is designed for the detection of 15 viral respiratory pathogens and four bacteria, including *Bordetella parapertussis*, *Bordetella pertussis*, *Chlamydophila pneumoniae*, and *Mycoplasma pneumoniae* [29]. It is a feasible and reliable tool for the estimation of the age-related prevalence of susceptible organisms [29,30]. The 300 μL nasopharyngeal swab samples were analyzed with the FilmArray^TM^ RP according to the manufacturer’s instructions. The FilmArray^TM^ RP combines three protocols: (i) nucleic acid extraction; (ii) nested multiplex PCR; and (iii) the interpretation of the results. The possible results of each target in a valid run were reported as detected or not detected. The overall procedures took approximately 1 h for a single test. Each run contained two controls. A qualitative result for each target was automatically interpreted by the software according to the endpoint melting curve data. A pathogen was recorded as detected if at least one of its corresponding assays was positive [31].

## 3. Results

**Figure 1:**
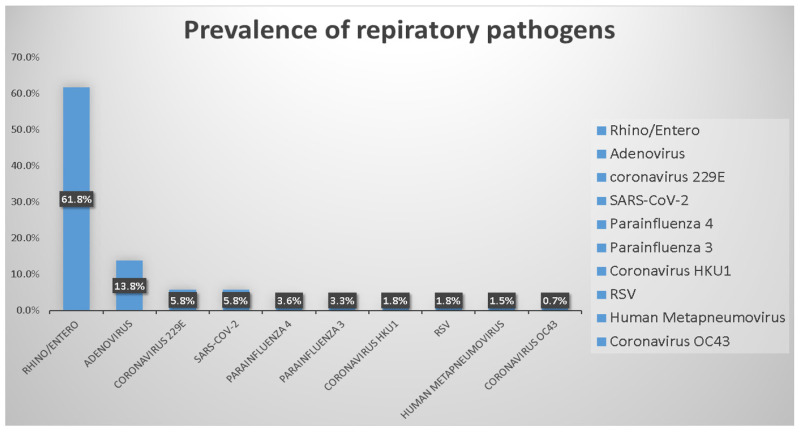
Prevalence of respiratory organisms detected by FilmArray^TM^ RP assay.

**Figure 2:**
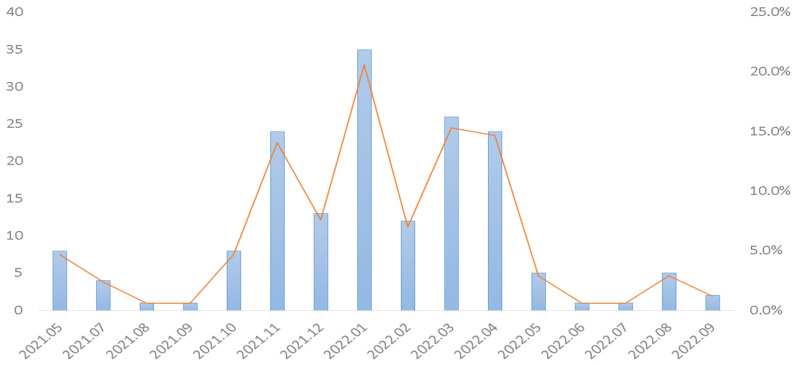
Monthly distribution of isolation rates of rhinovirus/enterovirus from emergency patients in central Taiwan from May 2021 to September 2022.

**Figure 3:**
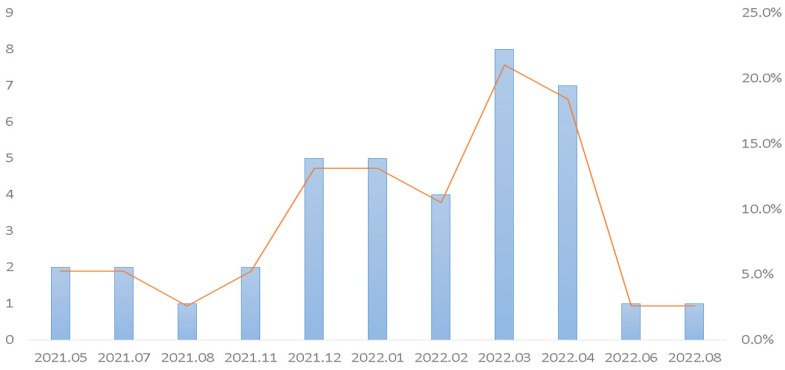
Monthly distribution of isolation rates of adenovirus from emergency patients in central Taiwan from May 2021 to September 2022.

Among the 804 specimens, 182 (22.6%, 182/804) had a single pathogen, 43 (5.3%, 43/804) had multiple pathogens, and 579 (72%, 579/804) had no pathogens. The overall positivity rate of the specimens was 27.9% (225/804). Rhinovirus/enterovirus was the most prevalent pathogen (61.8%, 170/275), followed by adenovirus (13.8%, 38/275), SARS-CoV-2 (5.8%, 16/275), and coronavirus 229E (5.8%, 16/275). The positivity rates of other pathogens were as follows: parainfluenza virus 4 (3.6%, 10/275); parainfluenza virus 3 (3.3%, 9/275); coronavirus HKU1 (1.8%, 5/275); respiratory syncytial virus (RSV) (1.8%, 5/275); human metapneumovirus (1.5%, 4/275), and coronavirus OC43 (0.7%, 2/275). The frequency distribution of pathogens is shown in Figure 1. Figure 2 shows that the test positivity rate of rhinovirus/enterovirus, and Figure 3 shows the test positivity rate of adenovirus. In the present study, the median age of these 182 patients was 4 years (0.3–90).

Among the 225 specimens, 43 (5.3%, 43/804) were positive for more than one pathogen. The largest proportion (41.9%, 18/43) of multiorganism-positive samples had combinations of adenovirus plus rhinovirus/enterovirus. The combination of rhinovirus/enterovirus plus parainfluenza virus 3 was the second most common combination, making up 11.6% (5/43) of all multiorganism-positive specimens. The combination of rhinovirus/enterovirus plus parainfluenza virus 4 was the third most common combination, making up 9.3% (4/43) of all multiorganism-positive specimens. The combination of rhinovirus/enterovirus plus respiratory syncytial virus and coronavirus 229E plus rhinovirus/enterovirus was the fourth most common types (7%, 3/43). In the present study, the median age of these 43 patients was 3 years (1–20). Table 1 summarizes the multiorganism-positive samples. Intriguingly, five patients had coinfections with three pathogens (11.6%, 5/43), and one patient had coinfections with four pathogens simultaneously (2.3%, 1/43). However, if four or more organisms are detected in a specimen, retesting is recommended to confirm the polymicrobial result according manufacturer’s instructions. Notably, the positivity rate of SARS-CoV-2 was 7.1% (16/225). The results for SARS-CoV-2 were confirmed by the cobas Liat system (Roche Molecular Systems, Inc., Branchburg, NJ, USA). All of the results were consistent with those of the reference method in the cobas Liat system. We found a mean Ct value of 20.4 (Table 2).

## 4. Discussion

The detection of respiratory viruses by multiplex PCR has been reported as a main diagnostic method for the identification of the nucleic acids of viral or bacterial organisms in respiratory tract infections. This is important for the detection of ARIs because the organisms can be present at low levels in clinical specimens, and their amount rapidly decreases over time despite the presence of symptoms. Our results provided the distributions of different pathogens in patients with symptoms of a respiratory infection at the emergency department. This study also provides new insights into the distribution of viral respiratory infections during the COVID-19 pandemic.

Multiple studies have shown that patients with respiratory-tract diseases commonly have more than one virus detected at frequencies as high as 35% [32,33,34]. Previously, Sreenath K et al. [8] showed that 47.1% of patients had SARS-CoV-2 coinfections with bacteria. *Klebsiella pneumoniae*, *staphylococcus aureus*, *haemophilus influenzae*, and *Streptococcus pneumoniae* were the main causes of bacterial coinfections. COVID-19 patients are often coinfected with other respiratory organisms, such as *pneumococcus* [10], *Mycoplasma pneumoniae* [35], *Legionella pneumophila* [36], cytomegalovirus [37], parainfluenza virus [38], respiratory syncytial virus [39], Epstein–Barr virus [40], rhinovirus [41], and influenza virus [9,10]. Recently, Motta JC et al. showed a patient with severe COVID-19 infection coinfected with adenovirus [42]. The authors indicated that the possibility of treatable organisms should be ruled out even in the case of a very rare coinfection, such as COVID-19 and adenovirus. In addition, Karaaslan A et al. found coinfections with other respiratory organisms in SARS-CoV-2-infected pediatric patients [43]. The authors identified a SARS-CoV-2-infected child who had two respiratory pathogens—rhinovirus/enterovirus and adenovirus—and the coinfection led to a considerably longer hospital stay. Our study also revealed that the SARS-CoV-2-infected pediatric patients were coinfected with adenovirus. One 2-year-old girl presented at the emergency department without any systemic disease and only suffered from intermittent fever. Orozoco-Hermandez et al. showed that a SARS-CoV-2-infected patient with coinfection with rhinovirus or enterovirus increased the severity of COVID-19 symptomatology, and eventually this patient progressed to multilobar pneumonia [44]. Additionally, Glass EL et al. reported that SARS-CoV-2 coinfection with rhinovirus was more frequent than coinfection with other respiratory viruses [45]. The authors suggested that patients coinfected with SARS-CoV-2 and rhinovirus were substantially more likely to have a cough than SARS-CoV-2-infected patients without coinfections. Peci A et al. revealed a small proportion (2.5%) of SARS-CoV-2-positive specimens coinfected with a seasonal respiratory virus using a laboratory-developed multiplex respiratory virus PCR method [46]. Our study also identified SARS-CoV-2-infected children who were coinfected with rhinovirus/enterovirus. One 1-year-old boy presented at the emergency department suffering from high fever.

Chen AP et al. indicated that rhinovirus appeared as a dominant circulating respiratory pathogen during a period of strengthened nonpharmaceutical interventions in Taiwan [47]. In this study, we showed that rhinovirus/enterovirus was the most prevalent pathogen (61.8%, 170/275) (Figure 1). Rhinovirus/enterovirus was detected here nearly every month in tropical countries [48]. Influenza A/B, enterovirus/rhinovirus, and respiratory syncytial virus (RSV) were most commonly detected in Singapore during the COVID-19 pandemic [49]. Multiple studies showed that rhinovirus/enterovirus is the most common detected virus in adult severe acute respiratory infections patients [50]. Chong YM et al. showed that the high prevalence of respiratory viruses in adults with severe acute respiratory infections was mainly attributed to rhinovirus/enterovirus [51]. In Taiwan, Sim YJ et al. demonstrated that rhinovirus/enterovirus and adenovirus were in almost persistent circulation during 2020 [52]. Indeed, our study showed that rhinovirus/enterovirus was in persistent circulation during the study period (Figure 2). Moreover, a recent study also showed that rhinovirus/enterovirus (32.7%) is the most common detected virus in Taiwanese population using BioFire FilmArray respiratory PCR panel 2.1 [53]. 

As previously described, adenovirus was in persistent circulation during 2020 when the incidence of COVID-19 was low [52]. Our results showed that adenovirus was the second most prevalent pathogen (13.8%, 38/275) (Figure 1). Figure 3 shows the adenovirus activity increased starting in approximately November 2021. In China, the prevalence of enteric adenovirus growing increasingly after nonpharmaceutical interventions had been declining in the post-COVID-19 period [54]. Li W et al. [55] showed that children aged 3–5 years old have the highest positive rate of adenovirus. The authors concluded that adenovirus infections significantly decreased in children during the COVID-19 pandemic. Similarly, we found that the highest positive rate of adenovirus was with the median age of 3 years old in the present study. 

Layman CP et al. showed that the FilmArray^TM^ system is feasible and reliable for use in an acute clinical setting. Their findings showed that the concordance between the FilmArray^TM^ system and viral culture was 94.5% [56]. Similarly, Lade H et al. indicated that the FilmArray^TM^ RP assay is easy to work and provide rapid identification of respiratory viruses [57]. Moreover, Tazi S et al. [31] showed that the estimated sensitivity and specificity of FilmArray, compared with the MAScIR SARS-CoV-2 M kit 2.0, were 100% and 79.2%, respectively. Recently, Livingstone et al. [58] showed that the application of FilmArray^TM^ RP 2.1 assay for COVID-19 remarkably reduced the time to obtain results spent on assessment cohort wards and the proportion of hospital-acquired COVID-19 infection. The authors concluded that the routine use of molecular point-of-care testing may become the standard of care in hospital admission procedures. Previously, our study showed that the cobas Liat SARS-CoV-2 and influenza A/B nucleic acid test is a feasible and reliable platform for the detection of SARS-CoV-2 [59]. Indeed, we showed that all results were consistent with those of the reference method in the cobas Liat system (Table 2). Berry GJ et al. [60] conducted a multicenter evaluation of BioFire RP2.1 for the detection of SARS-CoV-2. The authors concluded that the BioFire RP2.1 showed excellent performance in the detection of SARS-CoV-2. However, Chang YC et al. [53] reported that 42.6% of SARS-CoV-2 positive results obtained from FilmArray^TM^ RP 2.1 assay were inconsistent with other PCR systems (cobas Liat or cobas 6800 systems). They concluded that if the SARS-CoV-2 positive results by FilmArray^TM^ RP 2.1 assay should be reconfirmed by other quantitative RT-PCR assays. 

The main drawback of the present study is that we only retrospectively investigated the results of all the specimens to understand the incidence of each respiratory pathogen using the FilmArray^TM^ RP assay. We did not identify coinfecions involving specific pathogens by other methods, such as bacterial or viral culture tests in this study. Future studies should aim to overcome the drawbacks of this study by investigating relevant pathogens in coinfections. It is worth noting that our findings enhance our understanding of coinfections and provide new insight for the interpretation of coinfection detection arising from the FilmArray^TM^ RP assay in central Taiwan. 

## 5. Conclusions

In summary, the FilmArray^TM^ RP assay may be a feasible and reliable diagnostic tool for respiratory viruses in the emergency department during the COVID-19 pandemic. The FilmArray^TM^ RP assay may enhance clinical decision-making and limit the unnecessary use of antibiotics.

## Figures and Tables

**Table 1:** Summary of multiorganism-positive samples.

Pathogens	Percentage
Adenovirus + Rhino/Entero	41.9% (18/43)
Rhino/Entero + Parainfluenza 4	11.6% (5/43)
Rhino/Entero + Parainfluenza 3	9.3% (4/43)
Rhino/Entero + RSV	7% (3/43)
Coronavirus 229E + Rhino/Entero	7% (3/43)
Human Metapneumovirus+ Rhino/Entero	2.3% (1/43)
Parainfluenza 3 + Parainfluenza 4	2.3% (1/43)
SARS-CoV-2 + Adenovirus	2.3% (1/43)
SARS-CoV-2 + Rhino/Entero	2.3% (1/43)
Adenovirus + Coronavirus 229E + Rhino/Entero	4.7% (2/43)
Rhino/Entero + Parainfluenza 3 + Parainfluenza 4	2.3% (1/43)
Adenovirus + Rhino/Entero + Parainfluenza 3	2.3% (1/43)
Coronavirus HKU1 + Rhino/Entero + Parainfluenza 4	2.3% (1/43)
Human Metapneumovirus + Rhino/Entero + Parainfluenza 4 + RSV	2.3% (1/43)

**Table 2:** Detection of SARS-CoV-2 from nasopharyngeal swab (NPS) specimens using the FilmArray^TM^ RP 2.1 assay and cobas Liat system.

Age	Sex	FilmArray^TM^ RP 2.1 Assay	Cobas Liat System (Cycle Threshold Value)
1	F	detected	Positive (32.4)
1	M	detected	Positive (32.7)
7	M	detected	Positive (10.8)
6	F	detected	Positive (30.3)
1	M	detected	Positive (30.8)
6	F	detected	Positive (10.1)
6	M	detected	Positive (15.7)
3	F	detected	Positive (26.7)
8	M	detected	Positive (21.1)
2	F	detected	Positive (16.3)
6	F	detected	Positive (24.5)
2	F	detected	Positive (10.7)
3	F	detected	Positive (10.6)
2	M	detected	Positive (12)
2	F	detected	Positive (29.5)
1	M	detected	Positive (11.9)

## Data Availability

Not applicable.
